# The Roles of Notch Signaling in Liver Development and Disease

**DOI:** 10.3390/biom9100608

**Published:** 2019-10-14

**Authors:** Joshua M. Adams, Hamed Jafar-Nejad

**Affiliations:** 1 Department of Molecular and Human Genetics, Baylor College of Medicine, Houston, TX 77030, USA; jmadams@bcm.edu; 2 Program in Developmental Biology, Baylor College of Medicine, Houston, TX 77030, USA; 3 Medical Scientist Training Program, Baylor College of Medicine, Houston, TX 77030, USA

**Keywords:** notch signaling, liver, biliary development, liver fibrosis, liver regeneration, Alagille syndrome, genetic modifiers, liver metabolism

## Abstract

The Notch signaling pathway plays major roles in organ development across animal species. In the mammalian liver, Notch has been found critical in development, regeneration and disease. In this review, we highlight the major advances in our understanding of the role of Notch activity in proper liver development and function. Specifically, we discuss the latest discoveries on how Notch, in conjunction with other signaling pathways, aids in proper liver development, regeneration and repair. In addition, we review the latest in the role of Notch signaling in the pathogenesis of liver fibrosis and chronic liver disease. Finally, recent evidence has shed light on the emerging connection between Notch signaling and glucose and lipid metabolism. We hope that highlighting the major advances in the roles of Notch signaling in the liver will stimulate further research in this exciting field and generate additional ideas for therapeutic manipulation of the Notch pathway in liver diseases.

## 1. Introduction

Notch signaling plays critical roles in animal development, tissue homeostasis and human disease [1,2]. As a juxtacrine signaling mechanism mediated by interactions between transmembrane ligands and receptors, the Notch pathway allows neighboring cells to communicate and thereby regulates various processes, including apoptosis, proliferation, cell fate specification and asymmetric division [3]. Notch ligands presented by the signal-sending cells bind to the Notch receptors on the signal-receiving cells. Endocytosis of the ligand applies a pulling force to the Notch at the surface of the signal-receiving cell and results in a conformational change in the Notch receptor that exposes the cleavage site for ADAM10 metalloproteinase [2,4]. This is followed by cleavage by the γ-secretase complex, which releases the Notch intracellular domain (NICD). Finally, the NICD translocates to the nucleus and mediates the activation of Notch pathway targets together with the RBPJ (recombination signal binding protein for immunoglobulin kappa J region) and other co-factors [5].

Mammals have four Notch receptors called NOTCH1–4 and five canonical ligands called Delta-like 1 (DLL1), DLL3, DLL4, Jagged 1 (JAG1) and JAG2. Although there is some degree of redundancy between the function of individual Notch receptors and ligands, each component of the pathway also plays unique and non-redundant roles [6]. For example, the JAG1-NOTCH2 signaling axis has been widely known for its role in biliary specification and morphogenesis [7,8,9,10,11]. However, studies in vertebrate model organisms indicate that other Notch receptors and ligands also contribute to bile duct development [12,13].

In the liver, which is the subject of the current work, Notch signaling is important for the proper development of the biliary tree. This is evidenced by the bile duct paucity caused by *JAG1* and *NOTCH2* mutations in human patients with Alagille syndrome (ALGS) and the biliary abnormalities observed in vertebrate animal models with reduced Notch signaling [7,8,9,10,11,13,14,15,16,17]. The Notch pathway is also involved in liver regeneration and repair, liver fibrosis and metabolism [18]. Moreover, Notch signaling plays key roles in cancers of the liver [19,20]. Due to these broad and important actions, the contributions of Notch signaling to liver development and disease and the regulatory mechanisms for this pathway in the liver have been the subject of intense studies in recent years. These efforts have led to key advances in our understanding of the contributions of Notch signaling to biliary development and repair, liver regeneration and fibrosis and most recently liver metabolism. In this article, we will summarize the current state of the field in these areas, with a focus on more recent discoveries, and will suggest the questions that remain to be answered. Given the publication of multiple recent reviews on the role of Notch signaling in liver malignancies, this topic will not be covered here [21,22,23,24,25].

## 2. Notch in Bile Duct Development, Morphogenesis and Maintenance

**Figure 1:**
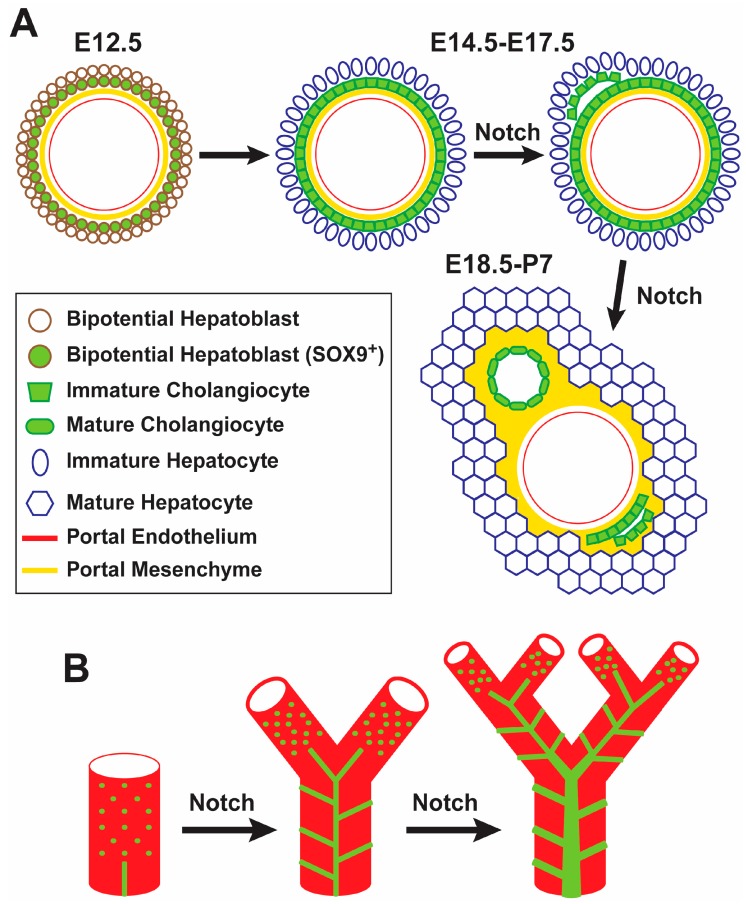
Notch signaling promotes biliary morphogenesis and the extension of the biliary tree. (**A**) A representation of cholangiocyte specification and biliary morphogenesis from E12.5 to the early postnatal period. (**B**) A three-dimensional (3D) representation of biliary development based on [17]. Differentiated cholangiocytes are first incorporated in a continuous homogeneous luminal network. This network is later reorganized into hierarchical tubular structures that form the adult biliary tree.

The Notch signaling pathway is critical for the proper maturation and morphogenesis of the intrahepatic biliary system. Among Notch-ligand pairs, the JAG1-NOTCH2 signaling axis is the one with an instrumental role in proper bile duct formation [10,11]. *Jag1* is expressed in portal mesenchymal cells, endothelial cells and biliary epithelial cells (BECs) [14]. As early as embryonic day 12.5 (E12.5)-E14.5, the mouse *Jag1* is expressed in the endothelium of the portal veins and the portal vein mesenchyme (PVM) [14,26]. JAG1 from PVM is thought to signal to surrounding bipotential hepatoblast cells, which express the Notch pathway targets HES1 and SOX9 and form a single-layered, cytokeratin-expressing “ductal plate” by E14.5-E15.5 [26,27]. Bile ducts later arise in some parts of the ductal plate, while the remaining, unincorporated cells become periportal hepatocytes and contribute to cholangiocytes lining smaller biliary conduits, which might serve as remnant hepatoblasts capable of activation upon severe or chronic injury (Figure 1A) [28]. Notch signaling is dispensable for the formation of the ductal plates [11,29]. However, upon conditional loss of Notch signaling in hepatoblasts, biliary morphogenesis is impaired [11,26,30,31]. Interestingly, while JAG1 presented by portal mesenchymal cells is essential for biliary development, loss of JAG1 in the endothelium or in BECs does not seem to result in abnormal biliary fate or morphology in mice [14,32]. These data indicate that Notch signaling is not necessary for the specification of BECs during liver development. However, reduced Notch signaling seems to impair the terminal differentiation of cholangiocytes and bile duct morphogenesis. It is also worth noting that ectopic activation of Notch signaling in the liver induces biliary cell fate specification and results in the formation of structures similar to bile ducts in the liver parenchyma [26,33,34], indicating that Notch activation is sufficient for biliary specification.

Based on studies in mice, a model has been proposed for the generation of bile ducts from ductal plates along the radial axis [26,27]. Based on this model, at the ductal plate, the developing bile ducts undergo a transitional asymmetric stage, at which the portal side of the developing bile duct consists of biliary cells, while the parenchymal side is still lined with hepatoblasts [26,27]. The transcription factor SOX9 also shows an asymmetric expression pattern during this transition (first expressed in the portal side of the developing duct, then in the parenchymal side) [27]. Moreover, liver-specific deletion of *Sox9* in *Alfp-Cre; Sox9^flox/flox^* mice results in a delay in the resolution of the asymmetric structures and in the expression of the biliary marker osteopontin (OPN) in nascent biliary cells [27]. However, by five weeks of age, the biliary system fully recovers in these animals [27]. These observations suggest that SOX9 is not essential for bile duct development but regulates its timing. *Sox9* is a direct target of Notch signaling in the liver and its expression in the liver is decreased in several Notch-deficient mouse models [9,14,26]. However, various genetic mouse models with decreased Notch signaling in the liver show bile duct abnormalities that persist into adulthood, unlike the *Sox9*-mutant biliary phenotype [9,10,14,15]. Therefore, a decrease in SOX9 expression cannot fully explain the bile duct morphogenesis defects in Notch-deficient livers, and other Notch pathway targets likely contribute to these phenotypes.

Given the elaborate three-dimensional (3D) structure of the biliary tree, conventional histological analysis of the biliary system is not able to fully determine the effect of specific genetic manipulations on the development of the biliary tree. To address this shortcoming, several groups have used elegant 3D visualization techniques, which involve filling the biliary tree with resin or ink, followed by light microscopy or X-ray microtomography of the liver [12,17,30,35,36,37,38]. Work in transgenic mice has provided strong evidence that Notch signaling regulates the density of the biliary tree in the liver periphery in a dosage-dependent manner [12]. When Notch signaling is disrupted early in hepatoblasts by *Albumin-Cre*-mediated deletion of *Notch2*, both *Notch2* and *Notch1*, or *Rbpj*, the peripheral biliary tree becomes less dense; when Notch signaling is overactivated by expressing N1-ICD with *Albumin-Cre*, the peripheral biliary tree becomes denser. Interestingly, *Albumin-Cre; Rbpj^flox/flox^* livers showed an initial reduction in the average number of bile ducts per portal vein up to postnatal day 60 (P60), but no further loss from P60 to P120 [36]. Based on this observation and other data, the authors suggested that in addition to its role in biliary development, Notch signaling might be required in the postnatal maintenance of a patent biliary tree [36].

In a more recent study, a new model has been proposed for the formation of the intrahepatic biliary tree by using 3D imaging techniques at multiple time points during embryonic development and postnatal growth of the liver (Figure 1B) [17]. According to this model, which to some extent challenges the idea of a single-layered and continuous ductal plate as the progenitor of bile ducts, discontinuous groups of cholangiocytes first appear around portal veins and then generate a fine, homogeneous structure called “continuous luminal network”. Later, dynamic rearrangement of this rather uniform network generates the hierarchical network that forms the biliary tree. As the liver grows, these two steps are continually repeated to extend the biliary tree towards the periphery [17]. Pharmacological inhibition of Notch signaling in early postnatal mice resulted in impaired elongation of the biliary tree, leading to the conclusion that Notch signaling is required for the formation of the continuous luminal network [17]. Of note, ink injection experiments suggest that the continuous luminal network persists in some parts of the biliary tree in P30 *Jag1* heterozygous livers [39], suggesting that Notch signaling also plays a role in reorganization of continuous luminal network to the mature, hierarchical biliary network (Figure 1B). It is worth mentioning that both groups reported that many of the existing OPN^+^ cells in these livers were not associated with ink-filled biliary structures. Altogether, these studies indicate that although biliary cell fate specification can occur in the absence of Notch signaling, proper biliary cell differentiation, bile duct morphogenesis in the radial axis, and the formation, growth and maintenance of the intrahepatic biliary tree all require Notch signaling.

The Notch signaling pathway acts in concert with other signaling pathways in the liver to direct proper biliary differentiation and development. As described in a recent review article, a complex regulatory network comprised of Notch, transforming growth factor beta (TGFβ), wingless-type MMTV integration site (WNT), fibroblast growth factor (FGF), bone morphogenetic protein (BMP) and Hippo signaling and a number of transcriptional regulators is involved in hepatoblast fate decision and expression of cholangiocyte versus hepatocyte genes in these cells [40]. In the remaining part of this section, we will highlight the connection between Notch signaling and two of these pathways, namely the TGFβ and the yes-associated protein (YAP)/transcriptional coactivator with PDZ-binding motif (TAZ) signaling pathways, in the context of biliary development and biliary tree formation.

Inhibition of TGFβ signaling with neutralizing antibodies during embryonic mouse development results in a reduction in the number of biliary cells around portal veins in E14.5 embryos [41]. Moreover, staining with a pan-cytokeratin antibody indicates that providing a local source of TGFβ to E12.5 mouse liver explants induces a gradient of biliary cytokeratin expression in the explants [41]. Similarly, treating bipotential mouse embryonic liver (BMEL) cells with TGFβ ligands in vitro results in the upregulation of cholangiocyte markers *Opn* and *Sox9* and downregulation of hepatocyte markers such as albumin and hepatic nuclear factor 4, alpha (*Hnf4a*) [27]. These observations indicate that a portal-to-parenchymal gradient of TGFβ signaling contributes to early stages of biliary development. It is worth noting that TGFβ signaling alone might not be essential for bile duct development, as evidenced by normal biliary tree formation in P32 animals in which TGFβ receptor II is conditionally knocked out in embryonic hepatoblasts [35].

**Figure 2:**
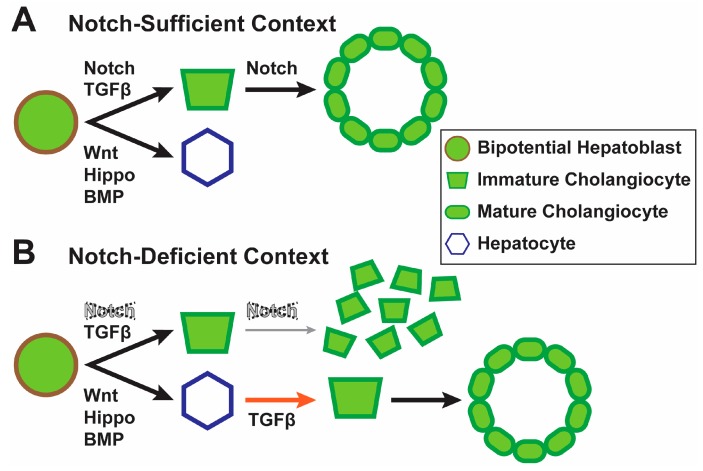
TGFβ promotes hepatocyte to cholangiocyte transdifferentiation in livers with severe Notch deficiency. (**A**) In a Notch-sufficient context, Notch and transforming growth factor beta (TGFβ) signaling help in specifying cholangiocytes and promoting the development of mature biliary structures. Wingless-type MMTV integration site (WNT), Hippo and bone morphogenetic protein (BMP) signaling promote hepatocyte fate. (**B**) In a severe Notch-deficient context, biliary morphogenesis is impaired and TGFβ signaling promotes transdifferentiation of hepatocytes to cholangiocytes (the orange arrow) to form patent biliary structures [35].

It was previously reported that mice with combined deletion of the Notch effector *Rbpj* and the transcription factor *Hnf6* in hepatoblasts (*Albumin-Cre; Hnf6^flox/flox^ Rbpj^flox/flox^*) initially show impaired biliary development, but exhibit a significant recovery and establish a functional biliary tree later in life [42]. This observation had suggested that a program entirely independent of Notch signaling can generate the biliary tree in adult mice if the Notch-dependent developmental program for this process fails. A recent study has shown that an unexpected compensatory role for TGFβ underlies this phenomenon [35]. The study also demonstrated that TGFβ signaling acted upon differentiated hepatocytes rather than immature cholangiocytes or hepatoblasts in populating the biliary tree in the adult. When TGFβ receptor 2 (*Tgfbr2*) was knocked out in hepatoblasts lacking *Rbpj* and *Hnf6*, intrahepatic bile ducts remained absent in older animals. An elegant set of experiments was used to demonstrate that activation of TGFβ signaling in hepatocytes induced their transdifferentiation to cholangiocytes capable of joining the extant biliary structures and formation of patent biliary conduits [35] (Figure 2). This study revealed a remarkable plasticity in adult hepatocytes, which allows them to transdifferentiate into cholangiocytes in a TGFβ-dependent manner and to restore a functional biliary tree in Notch-deficient livers. It also demonstrated a role for TGFβ in biliary morphogenesis beyond the embryonic and early postnatal biliary tree.

The Hippo signaling pathway is a major regulator of organ size in animals [43]. When Hippo signaling is active, its nuclear effector YAP/TAZ is phosphorylated and kept outside of the nucleus. Loss of Hippo signaling results in reduced YAP phosphorylation, which promotes YAP’s nuclear localization and activation of its downstream targets [43,44].

Conditional loss of Hippo pathway components in the liver results in significant liver overgrowth [45]. This is likely due to the increased activity of the Hippo pathway effector YAP/TAZ, because overexpression of a mutant version of YAP/TAZ with enhanced nuclear localization and transcriptional activity also results in a dramatic increase in liver size and in hepatocyte proliferation [46]. Notably, in addition to its role in proliferation, Hippo signaling also regulates cell fate in the liver. The YAP protein and its target CTGF are enriched in biliary cells in the adult liver [47]. Moreover, ectopic activation of YAP in mature hepatocytes results in their dedifferentiation to bipotential cells, indicating that Hippo signaling helps maintain the hepatocyte fate [47]. This study reported that YAP activation results in increased expression of *Jag1*, *Notch1* and *Notch2*, and that YAP is recruited to the *Notch2* regulatory region [47]. Moreover, simultaneous loss of Notch signaling in hepatocytes impaired the ability of overexpressed YAP to induce proliferation and dedifferentiation to a bipotential fate in these cells [47]. Together, these observations led to the conclusion that when Hippo signaling is compromised in mature hepatocytes, YAP acts through the Notch pathway to promote biliary fate.

A more recent study used *Lats1^–/–^; Albumin-Cre; Lats2^flox/flox^* mice to provide strong evidence that during liver development, activation of YAP/TAZ in the liver due to the loss of upstream Hippo pathway components LATS1 and LATS2 promotes the differentiation of bipotential hepatoblasts into BECs [44]. However, the effect of YAP/TAZ in this context was not correlated with increased *Notch2* expression and Notch pathway activity, but was instead associated with enhanced TGFβ signaling [44]. Moreover, the long-term effects of loss of Hippo signaling in this model were not inhibited by simultaneous loss of *Notch2*, were distinct from the phenotypes caused by activation of Notch signaling in hepatocytes, and were associated with activation of TGFβ signaling [44]. It remains to be seen whether epistasis analysis supports a direct role for TGFβ signaling in YAP-induced biliary fate specification and development. Regardless, future studies will show whether the discrepancy between these two studies is due to the difference between the models used to activate YAP/TAZ in hepatocytes or not.

## 3. The Role of Notch Signaling and Its Modifiers in the Pathogenesis of Liver Disease in Alagille Syndrome

Alagille syndrome (ALGS) is a multisystem developmental disorder affecting the biliary system, cardiovascular system, kidney, skeleton and other organs. Most ALGS patients have a dominant mutation in *JAG1* (~95%) or *NOTCH2* (~1–2%), and the genetic cause of the remaining cases is not known [7,8,48,49,50]. ALGS patients show extreme phenotypic variability in the severity of biliary defects and their progression with age [51,52]. However, there is no known genotype-phenotype correlation in ALGS [53,54,55,56]. Importantly, several studies have reported discordant phenotypes in monozygotic twins with ALGS [57,58,59], strongly suggesting a key role for the environment in determining the severity of the ALGS phenotypes. In addition, *Jag1* heterozygous phenotypes in mice are highly sensitive to genetic background and to the gene dosage of other Notch pathway components [9,10,60,61,62], suggesting that genetic modifiers are also likely to contribute to the ALGS phenotypic variability. Moreover, treatments for ALGS liver disease are limited and mainly supportive, with the only curative modality being liver transplantation [63,64]. Accordingly, identification of genetic modifiers of the disease phenotypes is critical for better and earlier assessment of prognosis and in developing novel treatment modalities. In this section, we will discuss several candidate genetic modifiers of the ALGS liver phenotypes that have been identified in mice and human patients.

Mutations in glycosyltransferase enzymes that add sugar residues to epidermal growth factor-like (EGF) repeats on Notch receptors and ligands are known to affect Notch signaling [65,66,67]. Dosage-sensitive genetic interactions in the context of biliary system have been reported between *Jag1* and enzymes responsible for addition of two distinct sugar residues to EGF repeats: fringe genes, whose protein products add *N*-acetylglucosamine (GlcNAc) to specific EGF repeats, and *Poglut1*, whose protein product adds *O*-linked glucose to specific EGF repeats.

The Fringe family of proteins, which in mammals consist of lunatic, radical and manic fringe (LFNG, RFNG, MFNG), are known to alter Notch signaling through their glycosyltransferase activity [68,69]. In a 2008 study, liver histology was analyzed in *Jag1^+/–^* animals that were simultaneously heterozygous for *Lfng*, *Mfng* or *Rfng*. While normal liver architecture was observed in single heterozygous animals for each of the fringe genes, *Jag1^+/–^; Rfng^+/–^* and *Jag1^+/–^; Lfng^+/–^* animals exhibited significant proliferation of biliary structures, which was not observed in control animals [61]. Notably, this proliferation was not accompanied by bile duct paucity. In contrast, *Jag1^+/–^; Mfng^+/–^* did not exhibit a proliferation of biliary structures like the other two double-heterozygous animals but showed a mild yet significant increase in bile duct density compared to control animals. The authors also reported normal hepatic and biliary structures in the livers of adult animals homozygous for each fringe gene. Together, these studies suggest that in *Jag1* heterozygous animals, some aspects of biliary development are sensitive to the fringe gene dosage. The single Fringe protein in *Drosophila* inhibits Notch signaling mediated by the JAG1 ortholog Serrate via decreasing the Serrate-Notch binging [68,70]. However, the effects of mammalian fringe proteins on JAG1-mediated signaling are potentially receptor-specific and have not been clearly established. For example, one study reported that both MFNG and LFNG reduce JAG1-NOTCH2 signaling by decreasing JAG1-NOTCH2 binding [71]; however, another showed an enhancement of JAG1-NOTCH2 signaling by LFNG [72]. Moreover, although all three fringe proteins enhance JAG1-NOTCH1 binding, MFNG and LFNG inhibit but RFNG appears to enhance JAG1-NOTCH1 signaling [73,74]. Therefore, the precise mechanisms underlying the observed genetic interactions remain to be identified.

When kept on a mixed genetic background, animals heterozygous for a null allele of *Jag1* called *Jag1^dDSL^* did not show any ALGS phenotypes other than eye abnormalities [60]. However, we found, rather serendipitously, that *Jag1^dDSL/+^* animals show significant bile duct paucity accompanied by a ductular reaction on a pure C57BL/6 background [9]. Previous studies had shown that loss of the *Drosophila Poglut1* (*rumi*) impairs Notch signaling, and that POGLUT1/Rumi promotes *Drosophila* Notch signaling by adding *O*-glucose residues to EGF repeats on the Notch receptor, not its ligands [75,76]. In contrast, genetic experiments in mice showed that in animals doubly heterozygous for *Jag1* and *Poglut1*, the liver histology is significantly improved, with a partial rescue of the bile duct paucity observed in *Jag1^dDSL/+^* animals [9]. In contrast to the *Drosophila* Serrate which does not have any POGLUT1 target sites, four of the JAG1 EGF repeats have experimentally-verified POGLUT1 target sites [9]. Moreover, conditional loss of *Poglut1* in vascular smooth muscle cells resulted in a post-transcriptional increase in JAG1 protein level in these cells [9], which are thought to be the source of JAG1 during bile duct development [14]. While this observation suggests increased JAG1 protein stabilization as a potential mechanism for the observed rescue, the precise mechanism through which POGLUT1 regulates JAG1-mediated signaling in the liver awaits further studies.

A more recent study from our group has demonstrated a role for SOX9 as a modifier of the liver phenotype in *Jag1^+/–^* mice. As mentioned before, in a Notch-sufficient condition, loss of both copies of *Sox9* results in a mild delay in biliary development that recovers by 5 weeks of age [27]. However, we found that conditional loss of one copy of *Sox9* by using the *Albumin-Cre* transgene in the context of *Jag1* heterozygosity worsens the *Jag1^+/–^* biliary phenotypes, as assessed by liver histology, serum chemistry and 3D visualization of the biliary tree [39]. Furthermore, removing both copies of *Sox9* in *Jag1^+/–^* livers further worsened the phenotypes and resulted in ~50% lethality by P25 [39]. In agreement with studies on other models of Notch deficiency in the liver [15,35,42], the *Jag1^+/–^* biliary defects showed significant improvement by age [39]. Again, simultaneous loss of one copy of *Sox9* prevented this improvement in *Jag1^+/–^* mice. These observations suggested that the level of SOX9 can play a major role in determining the severity and progression of *Jag1* haploinsufficient liver phenotypes. In agreement with these observations, the levels of SOX9 were found to be inversely correlated with the severity of liver disease in patients with ALGS. Importantly, increasing *Sox9* expression by *Albumin-Cre* resulted in improved cholangiocyte numbers and biliary development in *Jag1^+/–^* animals without any long-term adverse effects [39]. This suggested that not only is *Sox9* a dosage-sensitive modifier of the ALGS liver phenotype in mice, but increasing its expression in the liver may be a strategy to improve ALGS liver disease.

Another study directly assessed the genetic basis for the variability in ALGS liver phenotypes by performing a genome-wide association study comparing ALGS patients with known *JAG1* mutations which had mild versus severe liver phenotypes [77]. A single-nucleotide polymorphism (SNP) upstream of thrombospondin 2 (*THBS2*) was significantly associated with disease severity. The authors showed that *Thbs2*, which encodes a secreted protein, is expressed in BECs and in the portal mesenchyme of the mouse liver. *Thbs2*-null animals showed normal bile duct morphology and density, but increased micro-vascularization in the periportal areas of the adult liver and a higher number of hepatic arteries per portal vein in 1-week old animals. THBS2 was found to inhibit the binding of JAG1 to NOTCH2 receptor in vitro. Moreover, JAG1-mediated Notch signaling was enhanced in *Thbs2*-null stromal cells. In fibroblasts isolated from control human subjects, THBS2 protein was elevated in those carrying the identified allele, suggesting that the risk allele increases THBS2 expression. It is worth mentioning that in fibroblasts isolated from ALGS patients, no correlation was observed between the presence of the risk allele and the level of THBS2 mRNA and protein, which might be due to the low number of samples and the cell type analyzed. Together, these observations strongly suggest that level of THBS2 in the developing liver might be inversely correlated with the severity of the ALG1S phenotypes. It will be interesting to examine the effects of altering *Thbs2* gene dosage on the bile duct phenotypes caused by *Jag1* heterozygosity in mice.

## 4. Notch and Liver Regeneration and Repair

The liver has a tremendous capacity for regeneration following damage. Regeneration itself involves many different signaling pathways and the various cell types present in the adult liver [78]. Notch signaling has increasingly become of interest in understanding the processes involved in repairing and regenerating the mammalian liver. Following the loss or damage to liver tissue, hepatocytes, BECs and vascular-related cells must proliferate to restore liver structure and function. The role of Notch signaling in regeneration can be broken into three main areas, hepatocyte regeneration, biliary regeneration and neovascularization of the newly derived liver tissue.

While Notch signaling is known to be important in biliary regeneration, there have been some questions towards its role in hepatocyte regeneration [79,80]. The current evidence is mixed and suggests a complicated role for Notch in hepatocyte regeneration. A 2004 study showed that Notch pathway activity increases in hepatocytes following partial hepatectomy (PHx) in rats and that siRNA-mediated silencing of *Notch1* and *Jag1* two days before PHx results in reduced hepatocyte proliferation in the 2-4 days following the procedure [81]. However, the same study showed that Notch inhibition did not result in decreased weight at similar endpoints following PHx, suggesting compensatory mechanisms for loss of Notch activity. More recently, inhibition of Notch activity following PHx in rats was reported to result in impaired hepatocyte proliferation and regeneration along with disruptions to hepatocyte cell-cycle progression [82]. However, the situation is different when viewed from the perspective of Hepatic Progenitor Cells (HPCs). When Notch signaling is activated in HPCs in vitro, hepatocyte markers such as alpha fetoprotein are downregulated and biliary markers are upregulated, suggesting that the Notch activity prevents hepatocyte and promotes cholangiocyte fate [79]. In zebrafish and mouse, biliary cells are known to be capable of giving rise to hepatocytes upon severe loss of hepatocytes if the proliferation capacity of the remaining hepatocytes is impaired [83,84,85,86,87]. In such contexts, cholangiocytes are thought to dedifferentiate into bipotential HPCs, proliferate and then differentiate into hepatocytes [83,84]. Notch inhibition during zebrafish liver regeneration has recently been shown to enhance biliary to hepatocyte fate transformation, while Notch overactivation impairs this process [88]. Together, these data suggest that while Notch activity may play a role in hepatocyte proliferation during regeneration, the degree of Notch activity and whether the proliferative capacity of hepatocytes is affected or not are important factors in determining how Notch signaling affects hepatocyte regeneration.

The role of Notch signaling in biliary regeneration is more straightforward and better understood. One of the models used to induce biliary damage and study biliary regeneration is feeding animals with diet containing 3,5-diethoxycarbonyl-1,4-dihydrocollidine (DDC), a toxin that targets biliary cells and induces bile duct injury, ductular reactions and liver fibrosis [89]. In *Notch2* liver-specific knockout animals (*Albumin-Cre; Notch2^flox/–^*) that are treated with DDC, HPCs accumulate and are incapable of differentiating into cholangiocytes to restore biliary structure and function [80]. Moreover, biliary regeneration is impaired following PHx in postnatal Notch-ablated mice (*Mx-Cre; Rbpj^flox/flox^* treated with poly I:C to induce recombination), as evidenced by reduced CK19 expression, increased cholestasis and necrosis [79]. Despite this failed biliary differentiation, liver volume is restored and thus, hepatocyte proliferation and differentiation are not thought to be affected in this model. As previously noted, Notch overactivation in HPCs in vitro promotes biliary fate, preventing differentiation to hepatocytes [79]. A liver progenitor cell line called bipotential murine oval liver (BMOL) forms biliary tubular structures when grown on Matrigel [80]. Formation of these structures is significantly impaired upon Notch inhibition, suggesting a mechanism through which Notch activity promotes biliary restoration following damage [80]. Thus, Notch signaling in damaged liver pushes the cell fate toward cholangiocytes and promotes the formation of biliary structures.

Following PHx, vascularization is critical for the generation of a functional liver. Upon PHx, the newly formed hepatocytes generate avascular parenchymal islands, which subsequently signal to sinusoidal endothelial cells (LSECs) to induce their proliferation and recruitment to the newly formed liver tissue [90]. Conditional loss of *Rbpj* in adult mice with *Mx-Cre* transgene results in a highly efficient (>90%) deletion of the gene in endothelial cells and the liver [91]. Within 2–5 days of PHx, these animals did not show a major defect in regenerating hepatic lobules [91]. However, they showed a reduction in LSEC proliferation response, accompanied by LSEC degeneration and a sinusoidal obstruction syndrome [91]. These observations highlight the importance of Notch signaling in another aspect of the regeneration process. It is worth noting that by 10 days after PHx, *Rbpj*-deficient livers showed an almost complete regeneration, although not all aspects of liver function were restored [91]. Therefore, these data also provide another example of the remarkable capacity of the mouse liver to at least partially compensate for the loss of Notch signaling. 

## 5. Notch and the Development of Liver Fibrosis

Upon liver damage, hepatocytes and biliary cells must be regenerated to repair and rebuild lost tissue. In many organ systems, myofibroblasts secrete extracellular matrix (ECM) as part of a damage response. In the liver, quiescent Hepatic Stellate Cells (HSCs) are activated upon damage and differentiate to myofibroblast-like cells. These activated myofibroblasts deposit collagen-rich ECM during the wound healing response, to be resolved upon withdrawal of the insult and termination of tissue regeneration. If uncontrolled, this deposition can lead to fibrosis and impaired liver function [92,93]. Notch signaling has been suspected for some time to play a role in liver fibrosis [18]. Indeed, the observation that ALGS patients rarely go onto cirrhosis and exhibit less fibrotic deposition than other chronic liver disorders was an early clue toward a role for Notch signaling in the liver fibrotic response [18,94].

**Figure 3:**
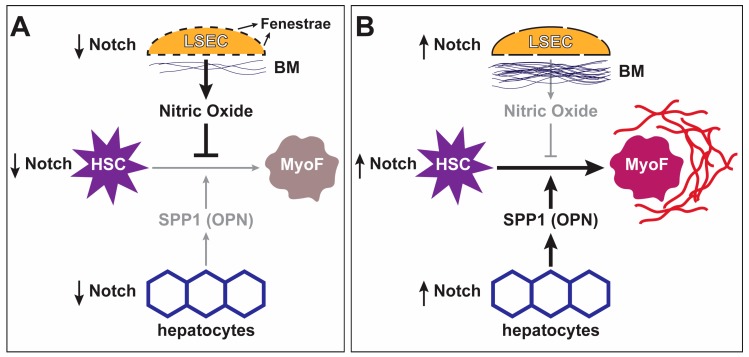
Notch signaling promotes Hepatic Stellate Cell (HSC) differentiation into myofibroblasts and liver fibrosis: (**A**) when Notch signaling is low, liver sinusoidal endothelial cells (LSECs) secrete nitric oxide, which inhibits the differentiation of hepatic stellate cells (HSCs) into myofibroblasts (MyoF). (**B**) Increased Notch activity in LSECs promotes their basement membrane (BM) production and reduces their fenestration and nitric oxide secretion, which in turn relieves nitric oxide’s inhibitory effect on HSC-to-myofibroblast differentiation. Additionally, increased Notch signaling in HSCs directly promotes their differentiation into myofibroblasts. Finally, activation of Notch signaling in hepatocytes promotes the secretion of SPP1 (OPN) by these cells, which also promotes myofibroblast differentiation. Myofibroblasts generated in response to increased Notch signaling promote liver fibrosis.

The mechanism and role for Notch signaling in liver fibrosis has been advanced over the past decade. Rat HSCs express Notch receptors in vitro and begin expressing JAG1 upon activation and differentiation to a myofibroblast-like cell [95]. Importantly, myofibroblast activation and subsequent collagen deposition in the lung has also been found to be partially dependent on Notch activity, suggesting that the role for Notch in fibrosis is not limited to the liver [96]. Carbon tetrachloride (CCl_4_) is a hepatotoxin commonly used to induce liver damage and fibrosis in rodent animal models. In vivo experimentation has shown that Notch inhibition in the context of CCL_4_-induced liver damage significantly impairs HSC activation and the development of fibrosis [97]. The in vivo dependence of HSC activation on Notch signaling suggests that inhibition of this pathway in the liver can prevent or significantly decrease fibrosis (Figure 3).

Direct activation of HSCs by Notch is not the only known mechanism through which Notch activity modulates fibrosis. Recent studies have demonstrated that Notch activation of LSECs and hepatocytes leads to subsequent HSC activation and fibrosis. LSECs are normally fenestrated, allowing the interaction of liver parenchyma with the sinusoidal space. Fenestrated LSECs secrete nitric oxide (NO) to maintain HSCs in a quiescent state [98]. Upon liver injury, LSECs are activated and develop a basement membrane, isolating the liver parenchyma and the sinusoidal spaces. Moreover, they reduce the secretion of NO and deposit ECM material [98]. NO has been shown to maintain HSCs in a quiescent state, while LSEC-derived ECM promotes HSC activation. Thus, reduction in NO and increased ECM deposition result in HSC activation and subsequent fibrotic deposition [98,99,100]. A recent study showed that Notch can lead to HSC activation by acting directly on LSECs [101]. Using the mouse as a model, the authors showed that endothelial-specific Notch activation resulted in the dedifferentiation and activation of LSECs, thereby leading to HSC activation and worsening of CCL_4_-induced liver fibrosis [101]. In another study, the role of Notch activity in hepatocytes on fibrosis was assessed. The authors showed that the number of HES1^+^ hepatocytes was increased in patients with more severe cases of nonalcoholic steatohepatitis (NASH) [102]. Moreover, the authors demonstrated that hepatocyte-specific Notch inhibition in a mouse model of NASH resulted in reduced fibrotic deposition and impaired HSC activation without affecting the degree of hepatocyte injury or liver inflammation [102]. Finally, the study suggested a mechanism whereby Notch-induced SOX9 expression in hepatocytes resulted in increased expression of OPN (official name: “secreted phosphoprotein 1” or *Spp1*), leading to HSC activation and the development of fibrosis. These two studies indicate that while Notch can directly activate HSCs to promote fibrosis, Notch activation in neighboring cell types can also result in HSC activation and the subsequent development of fibrosis (Figure 3).

Similar to biliary development and repair, during the development of liver fibrosis Notch intersects with other signaling pathways including TGFβ, Hedgehog (Hh) and Hippo. TGFβ has previously been shown to play a role in HSC activation and subsequent liver fibrosis [93]. Studies of the long non-coding RNA LFAR1 (lnc-LFAR1, liver fibrosis-associated lncRNA1) in the mouse liver demonstrated a crosstalk between TGFβ activation and Notch signaling in liver fibrosis [103]. Lnc-LFAR1 knock-down in the liver resulted in impaired TGFβ signaling and a reduction in HSC activation and fibrosis upon CCl_4_ administration. Lnc-LFAR1 knockdown also resulted in reduced *Notch2*, *Notch3*, *Hes1* and *Hey2* expression. The authors suggested that the observed reduction in Notch receptor expression is a result of impaired TGFβ signaling [103]. Indeed, lnc-LFAR1 was found to increase SMAD2/3 binding to the *Notch2* and *Notch3* regulatory regions, suggesting that TGFβ signaling might directly result in increased Notch activity. Another study demonstrated that increasing TGFβ expression in HSC cultures resulted in increased expression of *Jag1* and downstream Notch target genes [104]. Thus, TGFβ in the liver may help drive fibrosis in part by promoting Notch activity in HSCs.

The Hedgehog (Hh) signaling pathway has also been demonstrated to promote HSC activation and differentiation to myofibroblasts in the liver [105]. Using in vitro and in vivo evidence, a 2013 study showed that Hh and Notch signaling engage in a positive-feedback loop in HSCs during fibrotic reactions [106]. The authors reported that bile duct ligation (BDL) resulted in an increase in the mRNA levels of *Notch2* and *Jag1*, and Notch pathway targets *Hes1*, *Hey1*, *Hey2* and *HeyL*. Treating primary HSCs with the Hh signaling antagonist GDC-0449 resulted in a significant reduction in the expression of *Notch2*, *Jag1* and the Notch target genes. In the same cell type, pharmacological inhibition of Notch signaling by using the γ-secretase inhibitor DAPT (N-[N-(3,5-difluorophenacetyl-L-alanyl)]-S-phenyl-glycine t-butyl ester) resulted in decreased Hh-signaling. Moreover, mice with HSC-specific deletion of the Hh-pathway component smoothened showed a reduction in the level of fibrosis and Notch signaling following BDL. This work showed a positive-feedback loop whereby Hh and Notch stimulate each other in HSCs and together promote HSC-activation and subsequent fibrosis.

In normal liver, *Sox9* is a downstream target of Notch signaling and is specifically expressed in cholangiocytes, but not in other cell types [26,27]. However, a recent study demonstrated that in human patients with chronic hepatitis C infection and in mouse models of liver injury (CCl_4_ injection and BDL), SOX9 is also found in regenerative hepatocytes and activated HSCs [107]. The authors reported that increased SOX9 expression correlated with the severity of liver fibrosis in patients with chronic hepatitis C infection [107]. The authors further demonstrated a link between elevated SOX9 and the Hippo pathway in the development of fibrosis. Inhibition of YAP in CCl_4_ and BDL mouse models of liver fibrosis by injecting the specific YAP1:TEAD inhibitor verteporfin resulted in reduced SOX9 expression in HSCs. Moreover, genetic and cell culture experiments strongly suggest that the observed pro-fibrotic function of SOX9 arises from its expression in HSCs, not in cholangiocytes and hepatocytes. Together, this work and the above-mentioned references [101,102] suggest that Notch and YAP may activate SOX9 in different cell types, with both leading to the activation of HSCs and induction of fibrosis.

Notch activity therefore plays a direct role in the development of liver fibrosis. Notch activation in HSCs, neighboring hepatocytes and LSECs leads to fibrotic deposition. Notch also acts in concert with other signaling pathways in the activation of HSCs. In-depth understanding of Notch’s role in liver fibrosis by these lines of investigation might ultimately lead to improved therapies for decreasing the severity of liver fibrosis and potentially preventing fibrosis-induced liver failure in chronic liver disease.

## 6. Notch and Liver Metabolism

Notch signaling is important not only in the development and maintenance of liver tissue but also in one of the liver’s most important roles, metabolism. Liver metabolism and Notch can be broadly broken into two categories: glucose and lipid metabolism. In this section, we will summarize what is currently known on the complicated role of Notch signaling in both glucose and lipid metabolism and highlight the open questions in the field.

The Forkhead box-containing transcription factor 1, FOXO1, plays an important role in glucose metabolism. *Foxo1* heterozygosity reduces the diabetic burden in mouse models of metabolic syndrome [108]. In 2007, FOXO1 was shown to directly bind to RBPJ and lead to increased Notch signaling in myoblasts, thereby preventing their differentiation [109]. In this study, FOXO1 was found to aid in the clearance of corepressors and the recruitment of coactivators at the *Hes1* promoter. Therefore, through this interaction, FOXO1 was found to drive downstream Notch signaling and influence cellular differentiation. However, considering FOXO1’s critical role in metabolism, it remained to be seen whether the FOXO1-Notch interaction influenced cellular metabolism as well. To address this question, Pajvani et al. analyzed the effects of removing one copy of both *Notch1* and *Foxo1* on metabolic syndrome-related phenotypes caused by a high fat diet (HFD) [108]. The double-heterozygous animals showed increased (improved) insulin sensitivity with reduced serum glucose level compared to single-heterozygous and control animals [108]. The authors demonstrated a synergistic relationship between NOTCH1 and FOXO1 in the expression of downstream metabolic genes such as *G6pc*, and reported that *Rbpj* knockout in the liver improved insulin sensitivity in mice fed a HFD. Finally, *Notch1* gain of function mutations resulted in worsened insulin resistance and increased serum glucose levels, reminiscent of the phenotypes seen in patients with metabolic syndrome. These observations suggested that the interaction between Notch and FOXO1 also plays a key role in liver metabolism.

Recent conflicting evidence, however, has raised questions on the exact role that Notch signaling plays in glucose metabolism in the liver. In a 2013 study, a positive correlation between Notch activation and gluconeogenesis was identified in patients with insulin resistance [110]. Specifically, increased Notch activity was shown to be correlated with elevated Glucose-6-phosphatase (G6PC) and phosphoenolpyruvate carboxykinase (PCK1) enzyme levels. Patients with hyperglycemia were also found to have relatively high Notch activity in the liver as assessed by *HES1* expression. In contrast, a 2016 study reported that hepatocyte-specific deletion of *Notch1* resulted in decreased insulin sensitivity and increased serum glucose, similar to what is seen in metabolic syndrome [111]. The authors also noted that G6PC and Peripilipin-5 were elevated in these animals, thus increasing gluconeogenesis and fasting serum glucose. The authors provide several potential reasons underlying the apparent discrepancy between their results and Pajvani’s report [108]. For example, in the previous study, animals were germline double-heterozygous for *Notch1* and *Foxo1*, whereas this study deleted both copies of *Notch1* only in hepatocytes using *Albumin-Cre*. Further research is needed to better understand the role of Notch signaling in glucose metabolism, which might aid in designing future therapies for metabolic disorders.

We previously discussed the role of Notch signaling and LSECs on liver fibrosis. Hepatic inflammation promotes insulin resistance in metabolic syndrome, but the mechanism was largely unknown [112,113,114]. New research has demonstrated that hepatic inflammation in metabolic syndrome is mediated by LSECs. Using mice on an HFD, Miyachi et al. found that LSECs recruit Notch-ligand presenting myeloid cells to the liver, which in turn signal to hepatocytes to modulate glucose metabolism [115]. These myeloid cells were found to promote gluconeogenesis in hepatocytes, as evidenced by increased *G6pc* expression and serum glucose levels. This is likely to be directly mediated by Notch pathway activation, because 1) Notch signaling inhibition under HFD conditions resulted in reduced *G6pc* expression and 2) cell culture assays demonstrated that leukocytes in direct contact with hepatocytes induced increased *G6pc* and *Hes1* expression in hepatocytes, which was impaired upon γ-secretase inhibition. Thus, Notch ligands expressed by LSEC-recruited inflammatory cells can lead to increased gluconeogenesis in hepatocytes and thereby result in insulin resistance.

Notch signaling has also been found to play a key role in lipid metabolism in the liver. The mTOR pathway is known to activate lipogenesis in the liver through induction of *SREBP-1c* expression [116]. Experiments in mice indicated that Notch can activate mTOR and promote lipogenesis [117]. The authors found that steatosis was significantly reduced in HFD-fed mice with a liver-specific *Rbpj*-knockout (*Albumin-Cre; Rbpj^flox/flox^*). The authors then deleted *Rbpj* in mice simultaneously lacking *Foxo1* in the liver. Steatosis was also improved in these animals, suggesting that Notch-induced steatosis was FOXO1-independent. To elucidate the mechanism, the authors analyzed *Srebp1c* expression and mTOR signaling and found that both were reduced in *Rbpj*-deleted livers, suggesting that Notch signaling enhances mTOR signaling in the liver. Importantly, inhibition of mTOR was sufficient to impede Notch-induced fatty liver. Thus, Notch signaling acted in an mTOR-dependent, FOXO1-independent manner to increase lipogenesis and steatosis [117]. This is in contrast to glucose metabolism, where Notch and FOXO1 interact. The question remained whether this effect was solely the result of changes in lipogenesis or whether changes in lipid breakdown via fatty-acid oxidation contribute to the observed findings. To answer this question, a more recent study generated *Notch1*-deficient animals by transgenic expression of a *Notch1* antisense construct under the control of the mouse mammary tumor virus long terminal repeat promoter (*Notch1* antisense mouse [118].) The authors found that on a HFD, the *Notch1* antisense mice have reduced hepatic triacylglycerol levels compared to wild-type HFD-fed mice [118]. In addition, HFD was found to correlate with increased NOTCH1 expression in the liver, suggesting that Notch was responsive to lipid intake. Importantly, NOTCH1 inhibition in the *Notch1* antisense mouse resulted in increased expression of genes involved in fatty-acid oxidation, suggesting that Notch activity is involved in the breakdown of lipids as well as the previously reported lipogenesis pathways [117,118].

## 7. Conclusions and Future Directions

Notch signaling plays many roles in the development, repair and homeostasis of the liver. Disruptions in Notch signaling result in liver disorders such as Alagille syndrome, where a failure to properly form the biliary tree results in cholestasis and its sequela. Notch signaling, while important in the repair process, can also promote fibrotic deposition, leading to subsequent liver failure. Notch signaling has also been found to help regulate sugar and fat metabolism in the liver and may play a role in the pathophysiology of insulin resistance and metabolic syndrome.

The last few years have seen multiple advances in our understanding of Notch signaling regulation and its roles in the liver. Many signaling pathways are now known to intersect with Notch during liver development. This opens the question if these interactions are preserved in repair and regeneration. HSC activation is known to be regulated not only by Notch but also by TGFβ signaling, suggesting that pathway interaction may play a role in other repair/regenerative processes. What specific cell types are involved in these different signaling pathways is also relevant. Notch signaling in liver cells can be activated by ligands presented by the infiltrating immune cells, as demonstrated in studies of liver metabolism [115]. Is the same true with respect to HSC activation and subsequent fibrosis?

Another exciting area is elucidation of the genetic and perhaps environmental bases of the great phenotypic variation observed in Alagille syndrome presentation and prognosis, which are still largely unknown despite the identification of several candidate genetic modifiers [9,39,61,77]. Some of the important challenges in this area are to identify additional disease modifiers, to confirm that the genetic modifiers identified in mice contribute to phenotypic variability in human patients, to establish that the genetic variants identified in human genome-wide studies are responsible for phenotypic variability in patients, and to provide mechanistic insight into how each modifier affects Notch signaling, biliary development and disease pathophysiology. Our recent work has highlighted how changing the expression level of a single gene downstream of Notch signaling (*Sox9*) can dramatically affect biliary development in the context of Notch deficiency without having much of an effect in wild-type animals [39]. This observation suggests that when considering potential genetic modifiers of Notch signaling in Alagille syndrome and perhaps in other diseases of haploinsufficiency, one cannot limit the search to those genes which already have a significant phenotype in a wild-type context. Finally, given the reports on discordant phenotypes in monozygotic twins with ALGS [57,58], environmental factors are likely to contribute to the phenotypic variability in this disease, as was shown in an elegant study of gene-environment interaction in another Notch-related disease, namely congenital scoliosis [119].

Evidence of a role for Notch signaling in liver metabolism is quite interesting and recent. Notch signaling in hepatocytes plays important roles in proper sugar and fat metabolism in the liver. Of note, some studies have suggested that Notch signaling in cells other than hepatocytes can also affect liver metabolism [120,121]. Therefore, in addition to an intrinsic role in hepatocytes, a Notch-dependent cross-talk between hepatocytes and adjacent cells or even distant organs is likely to play a role in this process. Additional work on the cell-type specific roles of Notch signaling may help us understand the basis for discrepancies seen among some studies on Notch and liver metabolism and provide novel insight into the potential role of Notch in metabolic disorders.

## References

[1] Artavanis-Tsakonas S., Muskavitch M.A. (2010). Notch: The past, the present, and the future. Curr. Top.Dev. Biol..

[2] Siebel C., Lendahl U. (2017). Notch Signaling in Development, Tissue Homeostasis, and Disease. Physiol. Rev..

[3] Bray S.J. (2016). Notch signalling in context. Nat. Rev. Mol. Cell Biol..

[4] Lovendahl K.N., Blacklow S.C., Gordon W.R. (2018). The Molecular Mechanism of Notch Activation. Adv. Exp. Med. Biol..

[5] Kopan R., Ilagan M.X. (2009). The canonical Notch signaling pathway: Unfolding the activation mechanism. Cell.

[6] Kopan R., Chen S., Liu Z. (2014). Alagille, Notch, and robustness: Why duplicating systems does not ensure redundancy. Pediatr. Nephrol..

[7] Li L., Krantz I.D., Deng Y., Genin A., Banta A.B., Collins C.C., Qi M., Trask B.J., Kuo W.L., Cochran J. (1997). Alagille syndrome is caused by mutations in human Jagged1, which encodes a ligand for Notch1. Nat. Genet..

[8] Oda T., Elkahloun A.G., Pike B.L., Okajima K., Krantz I.D., Genin A., Piccoli D.A., Meltzer P.S., Spinner N.B., Collins F.S. (1997). Mutations in the human Jagged1 gene are responsible for Alagille syndrome. Nat. Genet..

[9] Thakurdas S.M., Lopez M.F., Kakuda S., Fernandez-Valdivia R., Zarrin-Khameh N., Haltiwanger R.S., Jafar-Nejad H. (2016). Jagged1 heterozygosity in mice results in a congenital cholangiopathy which is reversed by concomitant deletion of one copy of Poglut1 (Rumi). Hepatology.

[10] McCright B., Lozier J., Gridley T. (2002). A mouse model of Alagille syndrome: Notch2 as a genetic modifier of Jag1 haploinsufficiency. Development.

[11] Geisler F., Nagl F., Mazur P.K., Lee M., Zimber-Strobl U., Strobl L.J., Radtke F., Schmid R.M., Siveke J.T. (2008). Liver-specific inactivation of Notch2, but not Notch1, compromises intrahepatic bile duct development in mice. Hepatology.

[12] Sparks E.E., Huppert K.A., Brown M.A., Washington M.K., Huppert S.S. (2010). Notch signaling regulates formation of the three-dimensional architecture of intrahepatic bile ducts in mice. Hepatology.

[13] Zhang D., Gates K.P., Barske L., Wang G., Lancman J.J., Zeng X.I., Groff M., Wang K., Parsons M.J., Crump J.G. (2017). Endoderm Jagged induces liver and pancreas duct lineage in zebrafish. Nat. Commun..

[14] Hofmann J.J., Zovein A.C., Koh H., Radtke F., Weinmaster G., Iruela-Arispe M.L. (2010). Jagged1 in the portal vein mesenchyme regulates intrahepatic bile duct development: Insights into Alagille syndrome. Development.

[15] Andersson E.R., Chivukula I.V., Hankeova S., Sjoqvist M., Tsoi Y.L., Ramskold D., Masek J., Elmansuri A., Hoogendoorn A., Vazquez E. (2018). Mouse Model of Alagille Syndrome and Mechanisms of Jagged1 Missense Mutations. Gastroenterology.

[16] Lorent K., Yeo S.Y., Oda T., Chandrasekharappa S., Chitnis A., Matthews R.P., Pack M. (2004). Inhibition of Jagged-mediated Notch signaling disrupts zebrafish biliary development and generates multi-organ defects compatible with an Alagille syndrome phenocopy. Development.

[17] Tanimizu N., Kaneko K., Itoh T., Ichinohe N., Ishii M., Mizuguchi T., Hirata K., Miyajima A., Mitaka T. (2016). Intrahepatic bile ducts are developed through formation of homogeneous continuous luminal network and its dynamic rearrangement in mice. Hepatology.

[18] Geisler F., Strazzabosco M. (2015). Emerging roles of Notch signaling in liver disease. Hepatology.

[19] Zender S., Nickeleit I., Wuestefeld T., Sorensen I., Dauch D., Bozko P., El-Khatib M., Geffers R., Bektas H., Manns M.P. (2013). A critical role for notch signaling in the formation of cholangiocellular carcinomas. Cancer Cell.

[20] Villanueva A., Alsinet C., Yanger K., Hoshida Y., Zong Y., Toffanin S., Rodriguez-Carunchio L., Sole M., Thung S., Stanger B.Z. (2012). Notch signaling is activated in human hepatocellular carcinoma and induces tumor formation in mice. Gastroenterology.

[21] Wang N., Wang S., Li M.Y., Hu B.G., Liu L.P., Yang S.L., Yang S., Gong Z., Lai P.B.S., Chen G.G. (2018). Cancer stem cells in hepatocellular carcinoma: An overview and promising therapeutic strategies. Ther. Adv. Med. Oncol..

[22] Huang Q., Li J., Zheng J., Wei A. (2019). The Carcinogenic Role of the Notch Signaling Pathway in the Development of Hepatocellular Carcinoma. J. Cancer.

[23] Gil-Garcia B., Baladron V. (2016). The complex role of NOTCH receptors and their ligands in the development of hepatoblastoma, cholangiocarcinoma and hepatocellular carcinoma. Biol. Cell.

[24] Cigliano A., Wang J., Chen X., Calvisi D.F. (2017). Role of the Notch signaling in cholangiocarcinoma. Expert Opin. Ther. Targets.

[25] Lu J., Xia Y., Chen K., Zheng Y., Wang J., Lu W., Yin Q., Wang F., Zhou Y., Guo C. (2016). Oncogenic role of the Notch pathway in primary liver cancer. Oncol. Lett..

[26] Zong Y., Panikkar A., Xu J., Antoniou A., Raynaud P., Lemaigre F., Stanger B.Z. (2009). Notch signaling controls liver development by regulating biliary differentiation. Development.

[27] Antoniou A., Raynaud P., Cordi S., Zong Y., Tronche F., Stanger B.Z., Jacquemin P., Pierreux C.E., Clotman F., Lemaigre F.P. (2009). Intrahepatic bile ducts develop according to a new mode of tubulogenesis regulated by the transcription factor SOX9. Gastroenterology.

[28] Carpentier R., Suner R.E., van Hul N., Kopp J.L., Beaudry J.B., Cordi S., Antoniou A., Raynaud P., Lepreux S., Jacquemin P. (2011). Embryonic ductal plate cells give rise to cholangiocytes, periportal hepatocytes, and adult liver progenitor cells. Gastroenterology.

[29] Lozier J., McCright B., Gridley T. (2008). Notch signaling regulates bile duct morphogenesis in mice. PLoS ONE.

[30] Falix F.A., Weeda V.B., Labruyere W.T., Poncy A., de Waart D.R., Hakvoort T.B., Lemaigre F., Gaemers I.C., Aronson D.C., Lamers W.H. (2014). Hepatic Notch2 deficiency leads to bile duct agenesis perinatally and secondary bile duct formation after weaning. Dev. Biol..

[31] Kodama Y., Hijikata M., Kageyama R., Shimotohno K., Chiba T. (2004). The role of notch signaling in the development of intrahepatic bile ducts. Gastroenterology.

[32] Loomes K.M., Russo P., Ryan M., Nelson A., Underkoffler L., Glover C., Fu H., Gridley T., Kaestner K.H., Oakey R.J. (2007). Bile duct proliferation in liver-specific Jag1 conditional knockout mice: Effects of gene dosage. Hepatology.

[33] Jeliazkova P., Jors S., Lee M., Zimber-Strobl U., Ferrer J., Schmid R.M., Siveke J.T., Geisler F. (2013). Canonical Notch2 signaling determines biliary cell fates of embryonic hepatoblasts and adult hepatocytes independent of Hes1. Hepatology.

[34] Tchorz J.S., Kinter J., Muller M., Tornillo L., Heim M.H., Bettler B. (2009). Notch2 signaling promotes biliary epithelial cell fate specification and tubulogenesis during bile duct development in mice. Hepatology.

[35] Schaub J.R., Huppert K.A., Kurial S.N.T., Hsu B.Y., Cast A.E., Donnelly B., Karns R.A., Chen F., Rezvani M., Luu H.Y. (2018). De novo formation of the biliary system by TGFbeta-mediated hepatocyte transdifferentiation. Nature.

[36] Sparks E.E., Perrien D.S., Huppert K.A., Peterson T.E., Huppert S.S. (2011). Defects in hepatic Notch signaling result in disruption of the communicating intrahepatic bile duct network in mice. Dis. Model Mech..

[37] Kaneko K., Kamimoto K., Miyajima A., Itoh T. (2015). Adaptive remodeling of the biliary architecture underlies liver homeostasis. Hepatology.

[38] Poncy A., Antoniou A., Cordi S., Pierreux C.E., Jacquemin P., Lemaigre F.P. (2015). Transcription factors SOX4 and SOX9 cooperatively control development of bile ducts. Dev. Biol..

[39] Adams J.M., Huppert K.A., Castro E.C., Lopez M.F., Niknejad N., Subramanian S., Zarrin-Khameh N., Finegold M.J., Huppert S.S., Jafar-Nejad H. (2019). Sox9 is a modifier of the liver disease severity in a mouse model of Alagille syndrome. Hepatology.

[40] Gerard C., Tys J., Lemaigre F.P. (2017). Gene regulatory networks in differentiation and direct reprogramming of hepatic cells. Semin. Cell Dev. Biol..

[41] Clotman F., Jacquemin P., Plumb-Rudewiez N., Pierreux C.E., Van der Smissen P., Dietz H.C., Courtoy P.J., Rousseau G.G., Lemaigre F.P. (2005). Control of liver cell fate decision by a gradient of TGF beta signaling modulated by Onecut transcription factors. Genes Dev..

[42] Walter T.J., Vanderpool C., Cast A.E., Huppert S.S. (2014). Intrahepatic bile duct regeneration in mice does not require Hnf6 or Notch signaling through Rbpj. Am. J. Pathol..

[43] Halder G., Johnson R.L. (2011). Hippo signaling: Growth control and beyond. Development.

[44] Lee D.H., Park J.O., Kim T.S., Kim S.K., Kim T.H., Kim M.C., Park G.S., Kim J.H., Kuninaka S., Olson E.N. (2016). LATS-YAP/TAZ controls lineage specification by regulating TGFbeta signaling and Hnf4alpha expression during liver development. Nat. Commun..

[45] Lu L., Li Y., Kim S.M., Bossuyt W., Liu P., Qiu Q., Wang Y., Halder G., Finegold M.J., Lee J.S. (2010). Hippo signaling is a potent in vivo growth and tumor suppressor pathway in the mammalian liver. Proc. Natl Acad. Sci. USA.

[46] Camargo F.D., Gokhale S., Johnnidis J.B., Fu D., Bell G.W., Jaenisch R., Brummelkamp T.R. (2007). YAP1 increases organ size and expands undifferentiated progenitor cells. Curr. Biol..

[47] Yimlamai D., Christodoulou C., Galli G.G., Yanger K., Pepe-Mooney B., Gurung B., Shrestha K., Cahan P., Stanger B.Z., Camargo F.D. (2014). Hippo pathway activity influences liver cell fate. Cell.

[48] McDaniell R., Warthen D.M., Sanchez-Lara P.A., Pai A., Krantz I.D., Piccoli D.A., Spinner N.B. (2006). NOTCH2 mutations cause Alagille syndrome, a heterogeneous disorder of the notch signaling pathway. Am. J. Hum. Genet..

[49] Kamath B.M., Bauer R.C., Loomes K.M., Chao G., Gerfen J., Hutchinson A., Hardikar W., Hirschfield G., Jara P., Krantz I.D. (2012). NOTCH2 mutations in Alagille syndrome. J. Med. Genet..

[50] Gilbert M.A., Bauer R.C., Rajagopalan R., Grochowski C.M., Chao G., McEldrew D., Nassur J.A., Rand E.B., Krock B.L., Kamath B.M. (2019). Alagille syndrome mutation update: Comprehensive overview of JAG1 and NOTCH2 mutation frequencies and insight into missense variant classification. Hum. Mutat..

[51] Emerick K.M., Rand E.B., Goldmuntz E., Krantz I.D., Spinner N.B., Piccoli D.A. (1999). Features of Alagille syndrome in 92 patients: Frequency and relation to prognosis. Hepatology.

[52] Hoffenberg E.J., Narkewicz M.R., Sondheimer J.M., Smith D.J., Silverman A., Sokol R.J. (1995). Outcome of syndromic paucity of interlobular bile ducts (Alagille syndrome) with onset of cholestasis in infancy. J. Pediatr..

[53] Kamath B.M., Munoz P.S., Bab N., Baker A., Chen Z., Spinner N.B., Piccoli D.A. (2010). A longitudinal study to identify laboratory predictors of liver disease outcome in Alagille syndrome. J. Pediatr. Gastroenterol. Nutr..

[54] Krantz I.D., Colliton R.P., Genin A., Rand E.B., Li L., Piccoli D.A., Spinner N.B. (1998). Spectrum and frequency of jagged1 (JAG1) mutations in Alagille syndrome patients and their families. Am. J. Hum. Genet..

[55] Mouzaki M., Bass L.M., Sokol R.J., Piccoli D.A., Quammie C., Loomes K.M., Heubi J.E., Hertel P.M., Scheenstra R., Furuya K. (2016). Early life predictive markers of liver disease outcome in an International, Multicentre Cohort of children with Alagille syndrome. Liver Int..

[56] Gilbert M.A., Spinner N.B. (2017). Alagille syndrome: Genetics and Functional Models. Curr. Pathobiol. Rep..

[57] Izumi K., Hayashi D., Grochowski C.M., Kubota N., Nishi E., Arakawa M., Hiroma T., Hatata T., Ogiso Y., Nakamura T. (2016). Discordant clinical phenotype in monozygotic twins with Alagille syndrome: Possible influence of non-genetic factors. Am. J. Med. Genet. Part A.

[58] Kamath B.M., Krantz I.D., Spinner N.B., Heubi J.E., Piccoli D.A. (2002). Monozygotic twins with a severe form of Alagille syndrome and phenotypic discordance. Am. J. Med. Genet..

[59] Zhang Y., Xiang B., Yu X. (2019). A novel JAG1 mutation causing Alagille syndrome presenting with giant hepatic nodules and discordant phenotype in monozygotic twins. Med. Clin..

[60] Xue Y., Gao X., Lindsell C.E., Norton C.R., Chang B., Hicks C., Gendron-Maguire M., Rand E.B., Weinmaster G., Gridley T. (1999). Embryonic lethality and vascular defects in mice lacking the Notch ligand Jagged1. Hum. Mol. Genet..

[61] Ryan M.J., Bales C., Nelson A., Gonzalez D.M., Underkoffler L., Segalov M., Wilson-Rawls J., Cole S.E., Moran J.L., Russo P. (2008). Bile duct proliferation in Jag1/fringe heterozygous mice identifies candidate modifiers of the Alagille syndrome hepatic phenotype. Hepatology.

[62] Kiernan A.E., Li R., Hawes N.L., Churchill G.A., Gridley T. (2007). Genetic background modifies inner ear and eye phenotypes of jag1 heterozygous mice. Genetics.

[63] Kamath B.M., Loomes K.M., Piccoli D.A. (2010). Medical management of Alagille syndrome. J. Pediatr. Gastroenterol. Nutr..

[64] Kamath B.M., Schwarz K.B., Hadzic N. (2010). Alagille syndrome and liver transplantation. J. Pediatr. Gastroenterol. Nutr..

[65] Haltom A.R., Jafar-Nejad H. (2015). The multiple roles of epidermal growth factor repeat O-glycans in animal development. Glycobiology.

[66] Varshney S., Stanley P. (2018). Multiple roles for O-glycans in Notch signalling. FEBS Lett..

[67] Harvey B.M., Haltiwanger R.S. (2018). Regulation of Notch Function by O-Glycosylation. Adv. Exp. Med. Biol..

[68] Bruckner K., Perez L., Clausen H., Cohen S. (2000). Glycosyltransferase activity of Fringe modulates Notch-Delta interactions. Nature.

[69] Moloney D.J., Panin V.M., Johnston S.H., Chen J., Shao L., Wilson R., Wang Y., Stanley P., Irvine K.D., Haltiwanger R.S. (2000). Fringe is a glycosyltransferase that modifies Notch. Nature.

[70] Xu A., Haines N., Dlugosz M., Rana N.A., Takeuchi H., Haltiwanger R.S., Irvine K.D. (2007). In vitro reconstitution of the modulation of Drosophila Notch-ligand binding by Fringe. J. Biol. Chem..

[71] Shimizu K., Chiba S., Saito T., Kumano K., Takahashi T., Hirai H. (2001). Manic fringe and lunatic fringe modify different sites of the Notch2 extracellular region, resulting in different signaling modulation. J. Biol. Chem..

[72] Hicks C., Johnston S.H., diSibio G., Collazo A., Vogt T.F., Weinmaster G. (2000). Fringe differentially modulates Jagged1 and Delta1 signalling through Notch1 and Notch2. Nat. Cell Biol..

[73] Kakuda S., Haltiwanger R. (2017). Deciphering the Fringe-Mediated Notch Code: Identification of Activating and Inhibiting Sites Allowing Discrimination between Ligands. Dev. Cell.

[74] Yang L.T., Nichols J.T., Yao C., Manilay J.O., Robey E.A., Weinmaster G. (2005). Fringe glycosyltransferases differentially modulate Notch1 proteolysis induced by Delta1 and Jagged1. Mol. Biol. Cell.

[75] Leonardi J., Fernandez-Valdivia R., Li Y.D., Simcox A.A., Jafar-Nejad H. (2011). Multiple O-glucosylation sites on Notch function as a buffer against temperature-dependent loss of signaling. Development.

[76] Acar M., Jafar-Nejad H., Takeuchi H., Rajan A., Ibrani D., Rana N.A., Pan H., Haltiwanger R.S., Bellen H.J. (2008). Rumi is a CAP10 domain glycosyltransferase that modifies Notch and is required for Notch signaling. Cell.

[77] Tsai E.A., Gilbert M.A., Grochowski C.M., Underkoffler L.A., Meng H., Zhang X., Wang M.M., Shitaye H., Hankenson K.D., Piccoli D. (2016). THBS2 Is a Candidate Modifier of Liver Disease Severity in Alagille Syndrome. Cell. Mol. Gastroenterol. Hepatol..

[78] Forbes S.J., Newsome P.N. (2016). Liver regeneration–Mechanisms and models to clinical application. Nat. Rev. Gastroenterol. Hepatol..

[79] Lu J., Zhou Y., Hu T., Zhang H., Shen M., Cheng P., Dai W., Wang F., Chen K., Zhang Y. (2016). Notch Signaling Coordinates Progenitor Cell-Mediated Biliary Regeneration Following Partial Hepatectomy. Sci. Rep..

[80] Fiorotto R., Raizner A., Morell C.M., Torsello B., Scirpo R., Fabris L., Spirli C., Strazzabosco M. (2013). Notch signaling regulates tubular morphogenesis during repair from biliary damage in mice. J. Hepatol..

[81] Kohler C., Bell A.W., Bowen W.C., Monga S.P., Fleig W., Michalopoulos G.K. (2004). Expression of Notch-1 and its ligand Jagged-1 in rat liver during liver regeneration. Hepatology.

[82] Zhang F., Zhang J., Li X., Li B., Tao K., Yue S. (2018). Notch signaling pathway regulates cell cycle in proliferating hepatocytes involved in liver regeneration. J. Gastroenterol. Hepatol..

[83] Choi T.Y., Ninov N., Stainier D.Y., Shin D. (2014). Extensive conversion of hepatic biliary epithelial cells to hepatocytes after near total loss of hepatocytes in zebrafish. Gastroenterology.

[84] He J., Lu H., Zou Q., Luo L. (2014). Regeneration of liver after extreme hepatocyte loss occurs mainly via biliary transdifferentiation in zebrafish. Gastroenterology.

[85] Lu W.Y., Bird T.G., Boulter L., Tsuchiya A., Cole A.M., Hay T., Guest R.V., Wojtacha D., Man T.Y., Mackinnon A. (2015). Hepatic progenitor cells of biliary origin with liver repopulation capacity. Nat. Cell Biol..

[86] Raven A., Lu W.Y., Man T.Y., Ferreira-Gonzalez S., O’Duibhir E., Dwyer B.J., Thomson J.P., Meehan R.R., Bogorad R., Koteliansky V. (2017). Cholangiocytes act as facultative liver stem cells during impaired hepatocyte regeneration. Nature.

[87] He J., Chen J., Wei X., Leng H., Mu H., Cai P., Luo L. (2019). mTORC1 Signaling is Required for the Dedifferentiation from Biliary Cell to Bi-potential Progenitor Cell in Zebrafish Liver Regeneration. Hepatology.

[88] Russell J.O., Ko S., Monga S.P., Shin D. (2019). Notch Inhibition Promotes Differentiation of Liver Progenitor Cells into Hepatocytes via sox9b Repression in Zebrafish. Stem. Cells Int..

[89] Fickert P., Stoger U., Fuchsbichler A., Moustafa T., Marschall H.U., Weiglein A.H., Tsybrovskyy O., Jaeschke H., Zatloukal K., Denk H. (2007). A new xenobiotic-induced mouse model of sclerosing cholangitis and biliary fibrosis. Am. J. Pathol..

[90] Ross M.A., Sander C.M., Kleeb T.B., Watkins S.C., Stolz D.B. (2001). Spatiotemporal expression of angiogenesis growth factor receptors during the revascularization of regenerating rat liver. Hepatology.

[91] Wang L., Wang C.M., Hou L.H., Dou G.R., Wang Y.C., Hu X.B., He F., Feng F., Zhang H.W., Liang Y.M. (2009). Disruption of the transcription factor recombination signal-binding protein-Jkappa (RBP-J) leads to veno-occlusive disease and interfered liver regeneration in mice. Hepatology.

[92] Klingberg F., Hinz B., White E.S. (2013). The myofibroblast matrix: Implications for tissue repair and fibrosis. J. Pathol..

[93] Tsuchida T., Friedman S.L. (2017). Mechanisms of hepatic stellate cell activation. Nat. Rev. Gastroenterol. Hepatol..

[94] Fabris L., Cadamuro M., Guido M., Spirli C., Fiorotto R., Colledan M., Torre G., Alberti D., Sonzogni A., Okolicsanyi L. (2007). Analysis of liver repair mechanisms in Alagille syndrome and biliary atresia reveals a role for notch signaling. Am. J. Pathol..

[95] Sawitza I., Kordes C., Reister S., Haussinger D. (2009). The niche of stellate cells within rat liver. Hepatology.

[96] Liu T., Hu B., Choi Y.Y., Chung M., Ullenbruch M., Yu H., Lowe J.B., Phan S.H. (2009). Notch1 signaling in FIZZ1 induction of myofibroblast differentiation. Am. J. Pathol..

[97] Chen Y., Zheng S., Qi D., Zheng S., Guo J., Zhang S., Weng Z. (2012). Inhibition of Notch signaling by a gamma-secretase inhibitor attenuates hepatic fibrosis in rats. PLoS ONE.

[98] Greuter T., Shah V.H. (2016). Hepatic sinusoids in liver injury, inflammation, and fibrosis: New pathophysiological insights. J. Gastroenterol..

[99] Langer D.A., Das A., Semela D., Kang-Decker N., Hendrickson H., Bronk S.F., Katusic Z.S., Gores G.J., Shah V.H. (2008). Nitric oxide promotes caspase-independent hepatic stellate cell apoptosis through the generation of reactive oxygen species. Hepatology.

[100] DeLeve L.D., Wang X., Guo Y. (2008). Sinusoidal endothelial cells prevent rat stellate cell activation and promote reversion to quiescence. Hepatology.

[101] Duan J.L., Ruan B., Yan X.C., Liang L., Song P., Yang Z.Y., Liu Y., Dou K.F., Han H., Wang L. (2018). Endothelial Notch activation reshapes the angiocrine of sinusoidal endothelia to aggravate liver fibrosis and blunt regeneration in mice. Hepatology.

[102] Zhu C., Kim K., Wang X., Bartolome A., Salomao M., Dongiovanni P., Meroni M., Graham M.J., Yates K.P., Diehl A.M. (2018). Hepatocyte Notch activation induces liver fibrosis in nonalcoholic steatohepatitis. Sci. Transl. Med..

[103] Zhang K., Han X., Zhang Z., Zheng L., Hu Z., Yao Q., Cui H., Shu G., Si M., Li C. (2017). The liver-enriched lnc-LFAR1 promotes liver fibrosis by activating TGFbeta and Notch pathways. Nat. Commun..

[104] Aimaiti Y., Jin X., Wang W., Chen Z., Li D. (2018). TGF-beta1 signaling regulates mouse hepatic stellate cell differentiation via the Jagged1/Notch pathway. Life Sci..

[105] Choi S.S., Omenetti A., Witek R.P., Moylan C.A., Syn W.K., Jung Y., Yang L., Sudan D.L., Sicklick J.K., Michelotti G.A. (2009). Hedgehog pathway activation and epithelial-to-mesenchymal transitions during myofibroblastic transformation of rat hepatic cells in culture and cirrhosis. Am. J. Physiol. Gastrointest. Liver Physiol..

[106] Xie G., Karaca G., Swiderska-Syn M., Michelotti G.A., Kruger L., Chen Y., Premont R.T., Choi S.S., Diehl A.M. (2013). Cross-talk between Notch and Hedgehog regulates hepatic stellate cell fate in mice. Hepatology.

[107] Athwal V.S., Pritchett J., Llewellyn J., Martin K., Camacho E., Raza S.M., Phythian-Adams A., Birchall L.J., Mullan A.F., Su K. (2017). SOX9 predicts progression toward cirrhosis in patients while its loss protects against liver fibrosis. EMBO Mol. Med..

[108] Pajvani U.B., Shawber C.J., Samuel V.T., Birkenfeld A.L., Shulman G.I., Kitajewski J., Accili D. (2011). Inhibition of Notch signaling ameliorates insulin resistance in a FoxO1-dependent manner. Nat. Med..

[109] Kitamura T., Kitamura Y.I., Funahashi Y., Shawber C.J., Castrillon D.H., Kollipara R., DePinho R.A., Kitajewski J., Accili D. (2007). A Foxo/Notch pathway controls myogenic differentiation and fiber type specification. J. Clin. Invest..

[110] Valenti L., Mendoza R.M., Rametta R., Maggioni M., Kitajewski C., Shawber C.J., Pajvani U.B. (2013). Hepatic notch signaling correlates with insulin resistance and nonalcoholic fatty liver disease. Diabetes.

[111] Bernsmeier C., Dill M.T., Provenzano A., Makowska Z., Krol I., Muscogiuri G., Semela D., Tornillo L., Marra F., Heim M.H. (2016). Hepatic Notch1 deletion predisposes to diabetes and steatosis via glucose-6-phosphatase and perilipin-5 upregulation. Lab. Invest..

[112] Osborn O., Olefsky J.M. (2012). The cellular and signaling networks linking the immune system and metabolism in disease. Nat. Med..

[113] Oh D.Y., Morinaga H., Talukdar S., Bae E.J., Olefsky J.M. (2012). Increased Macrophage Migration into Adipose Tissue in Obese Mice. Diabetes.

[114] Talukdar S., Oh D.Y., Bandyopadhyay G., Li D., Xu J., McNelis J., Lu M., Li P., Yan Q., Zhu Y. (2012). Neutrophils mediate insulin resistance in mice fed a high-fat diet through secreted elastase. Nat. Med..

[115] Miyachi Y., Tsuchiya K., Komiya C., Shiba K., Shimazu N., Yamaguchi S., Deushi M., Osaka M., Inoue K., Sato Y. (2017). Roles for Cell-Cell Adhesion and Contact in Obesity-Induced Hepatic Myeloid Cell Accumulation and Glucose Intolerance. Cell. Rep..

[116] Li S., Brown M.S., Goldstein J.L. (2010). Bifurcation of insulin signaling pathway in rat liver: mTORC1 required for stimulation of lipogenesis, but not inhibition of gluconeogenesis. Proc. Natl. Acad. Sci. USA.

[117] Pajvani U.B., Qiang L., Kangsamaksin T., Kitajewski J., Ginsberg H.N., Accili D. (2013). Inhibition of Notch uncouples Akt activation from hepatic lipid accumulation by decreasing mTorc1 stability. Nat. Med..

[118] Song N.J., Yun U.J., Yang S., Wu C., Seo C.R., Gwon A.R., Baik S.H., Choi Y., Choi B.Y., Bahn G. (2016). Notch1 deficiency decreases hepatic lipid accumulation by induction of fatty acid oxidation. Sci. Rep..

[119] Sparrow D.B., Chapman G., Smith A.J., Mattar M.Z., Major J.A., O’Reilly V.C., Saga Y., Zackai E.H., Dormans J.P., Alman B.A. (2012). A mechanism for gene-environment interaction in the etiology of congenital scoliosis. Cell.

[120] Jabs M., Rose A.J., Lehmann L.H., Taylor J., Moll I., Sijmonsma T.P., Herberich S.E., Sauer S.W., Poschet G., Federico G. (2018). Inhibition of Endothelial Notch Signaling Impairs Fatty Acid Transport and Leads to Metabolic and Vascular Remodeling of the Adult Heart. Circulation.

[121] Rubio-Aliaga I., Przemeck G.K., Fuchs H., Gailus-Durner V., Adler T., Hans W., Horsch M., Rathkolb B., Rozman J., Schrewe A. (2009). Dll1 haploinsufficiency in adult mice leads to a complex phenotype affecting metabolic and immunological processes. PLoS ONE.

